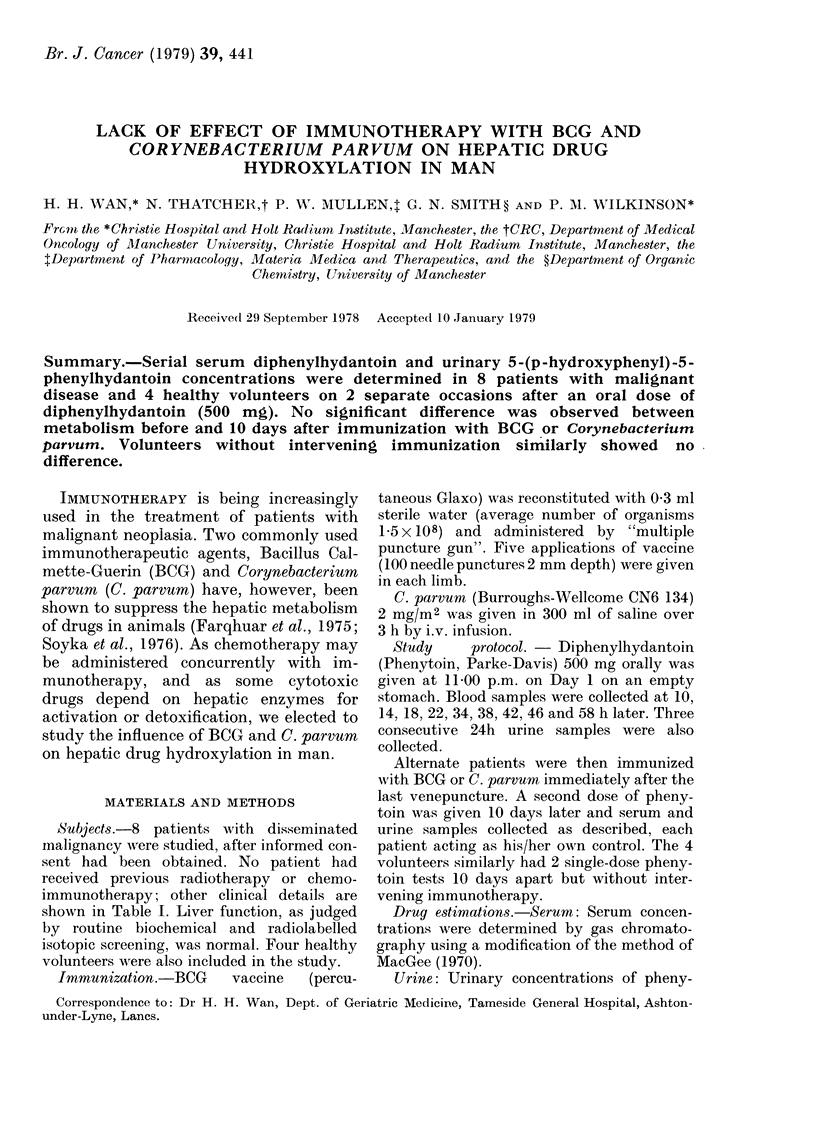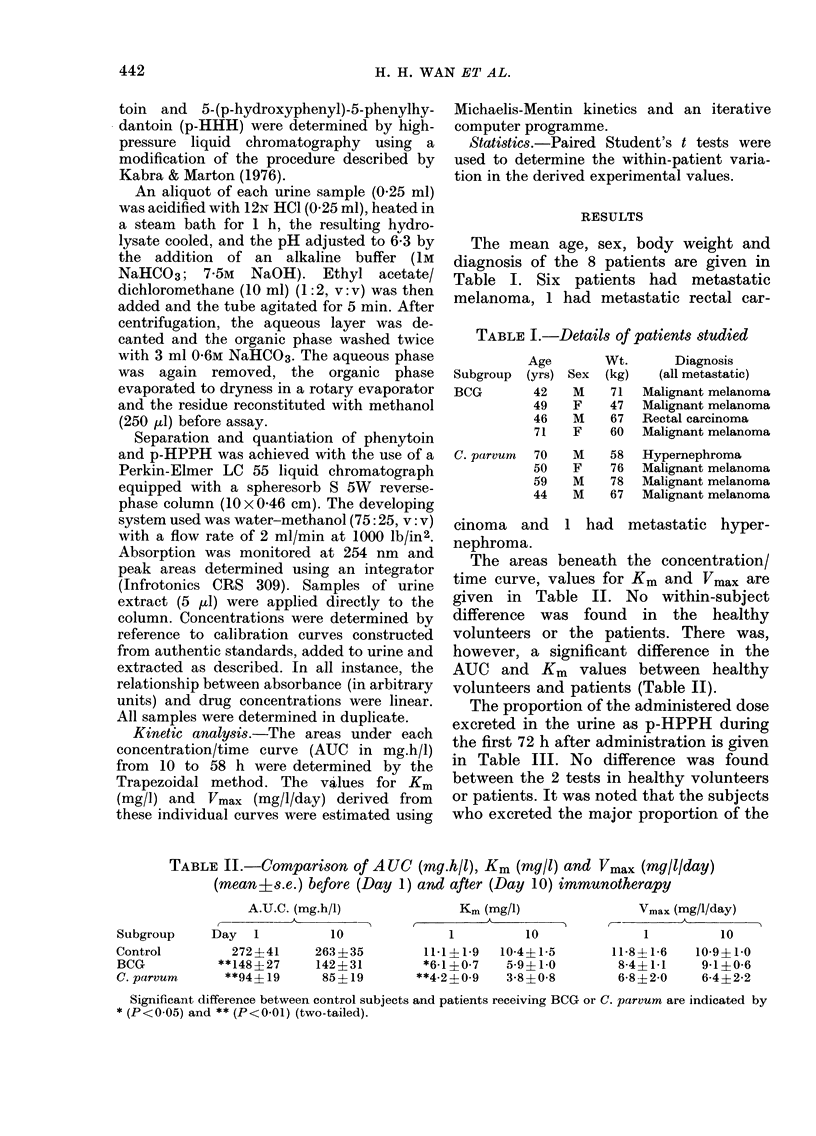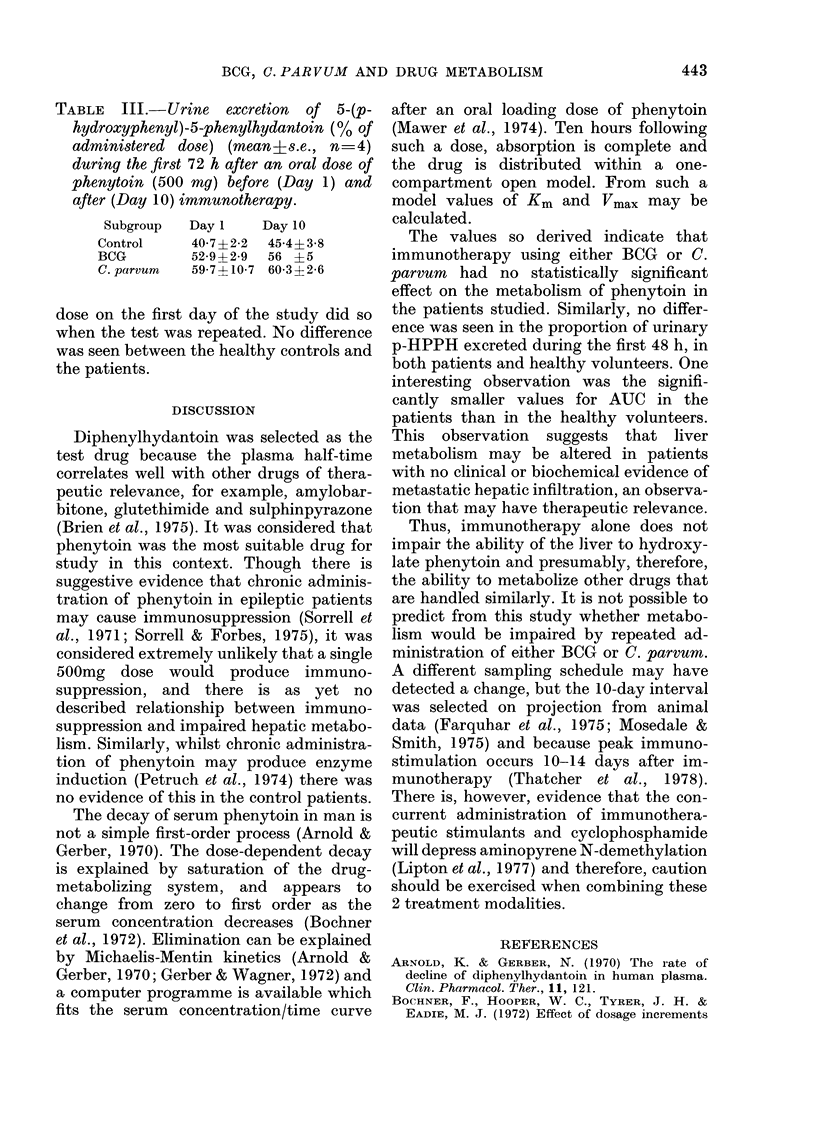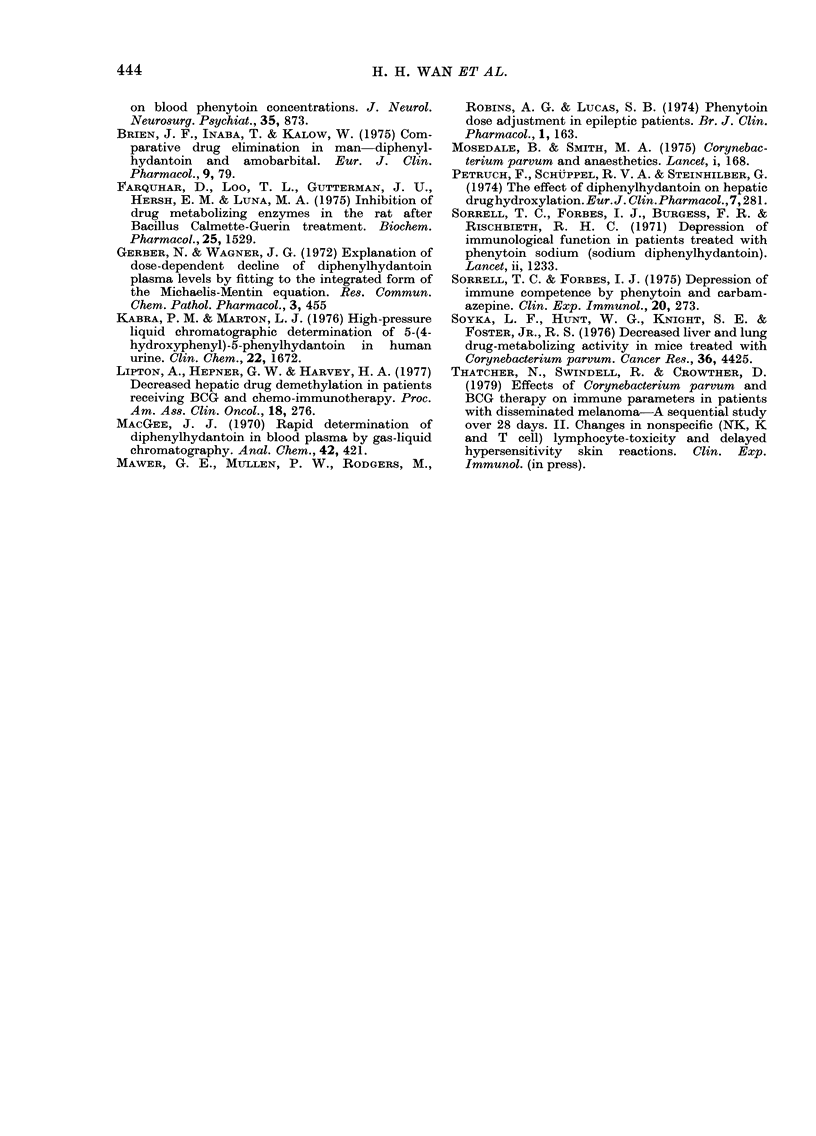# Lack of effect of immunotherapy with BCG and Corynebacterium parvum on hepatic drug hydroxylation in man.

**DOI:** 10.1038/bjc.1979.78

**Published:** 1979-04

**Authors:** H. H. Wan, N. Thatcher, P. W. Mullen, G. N. Smith, P. M. Wilkinson

## Abstract

Serial serum diphenylhydantoin and urinary 5-(p-hydroxphenyl)-5-phenylhydantoin concentrations were determined in 8 patients with malignant disease and 4 healthy volunteers on 2 separate occasions after an oral dose of diphenylhydantoin (500 mg). No significant difference was observed between metabolism before and 10 days after immunization with BCG or Corynebacterium parvum. Volunteers without intervening immunization similarly showed no difference.


*Br. J. Cancer* (1979) **39**, 441

### LACK OF EFFECT OF IMMUNOTHERAPY WITH BCG AND *CORYNEBACTERIUM PARVUM* ON HEPATIC DRUG HYDROXYLATION IN MAN

H. H. WAN,* N. THATCHER,† P. W. MULLEN,‡ G. N. SMITH§ AND P. M. WILKINSON*

*From the *Christie Hospital and Holt Radium Institute, Manchester, the †CRC, Department of Medical Oncology of Manchester University, Christie Hospital and Holt Radium Institute, Manchester, the ‡Department of Pharmacology, Materia Medica and Therapeutics, and the §Department of Organic Chemistry, University of Manchester*

Received 29 September 1978 Accepted 10 January 1979

**Summary.—Serial serum diphenylhydantoin and urinary 5-(p-hydroxyphenyl)-5-phenylhydantoin concentrations were determined in 8 patients with malignant disease and 4 healthy volunteers on 2 separate occasions after an oral dose of diphenylhydantoin (500 mg). No significant difference was observed between metabolism before and 10 days after immunization with BCG or *Corynebacterium parvum*. Volunteers without intervening immunization similarly showed no difference.**

IMMUNOTHERAPY is being increasingly used in the treatment of patients with malignant neoplasia. Two commonly used immunotherapeutic agents, Bacillus Calmette-Guerin (BCG) and *Corynebacterium parvum* (*C. parvum*) have, however, been shown to suppress the hepatic metabolism of drugs in animals (Farqhuar *et al.*, 1975; Soyka *et al.*, 1976). As chemotherapy may be administered concurrently with immunotherapy, and as some cytotoxic drugs depend on hepatic enzymes for activation or detoxification, we elected to study the influence of BCG and *C. parvum* on hepatic drug hydroxylation in man.

### MATERIALS AND METHODS

*Subjects.*—8 patients with disseminated malignancy were studied, after informed consent had been obtained. No patient had received previous radiotherapy or chemoimmunotherapy; other clinical details are shown in Table I. Liver function, as judged by routine biochemical and radiolabelled isotopic screening, was normal. Four healthy volunteers were also included in the study.

*Immunization.*—BCG vaccine (percutaneous Glaxo) was reconstituted with 0·3 ml sterile water (average number of organisms $1{\cdot}5 \times 10^8$) and administered by "multiple puncture gun". Five applications of vaccine (100 needle punctures 2 mm depth) were given in each limb.

*C. parvum* (Burroughs-Wellcome CN6 134) 2 mg/m$^2$ was given in 300 ml of saline over 3 h by i.v. infusion.

*Study protocol.* — Diphenylhydantoin (Phenytoin, Parke-Davis) 500 mg orally was given at 11·00 p.m. on Day 1 on an empty stomach. Blood samples were collected at 10, 14, 18, 22, 34, 38, 42, 46 and 58 h later. Three consecutive 24h urine samples were also collected.

Alternate patients were then immunized with BCG or *C. parvum* immediately after the last venepuncture. A second dose of phenytoin was given 10 days later and serum and urine samples collected as described, each patient acting as his/her own control. The 4 volunteers similarly had 2 single-dose phenytoin tests 10 days apart but without intervening immunotherapy.

*Drug estimations.—Serum*: Serum concentrations were determined by gas chromatography using a modification of the method of MacGee (1970).

*Urine*: Urinary concentrations of pheny-

Correspondence to: Dr H. H. Wan, Dept. of Geriatric Medicine, Tameside General Hospital, Ashton-under-Lyne, Lancs.

toin and 5-(p-hydroxyphenyl)-5-phenylhydantoin (p-HHH) were determined by high-pressure liquid chromatography using a modification of the procedure described by Kabra & Marton (1976).

An aliquot of each urine sample (0·25 ml) was acidified with 12N HCl (0·25 ml), heated in a steam bath for 1 h, the resulting hydrolysate cooled, and the pH adjusted to 6·3 by the addition of an alkaline buffer (1M $NaHCO_3$; 7·5M NaOH). Ethyl acetate/dichloromethane (10 ml) (1:2, v:v) was then added and the tube agitated for 5 min. After centrifugation, the aqueous layer was decanted and the organic phase washed twice with 3 ml 0·6M $NaHCO_3$. The aqueous phase was again removed, the organic phase evaporated to dryness in a rotary evaporator and the residue reconstituted with methanol (250 μl) before assay.

Separation and quantiation of phenytoin and p-HPPH was achieved with the use of a Perkin-Elmer LC 55 liquid chromatograph equipped with a spheresorb S 5W reverse-phase column (10×0·46 cm). The developing system used was water–methanol (75:25, v:v) with a flow rate of 2 ml/min at 1000 lb/in$^2$. Absorption was monitored at 254 nm and peak areas determined using an integrator (Infrotonics CRS 309). Samples of urine extract (5 μl) were applied directly to the column. Concentrations were determined by reference to calibration curves constructed from authentic standards, added to urine and extracted as described. In all instance, the relationship between absorbance (in arbitrary units) and drug concentrations were linear. All samples were determined in duplicate.

*Kinetic analysis.*—The areas under each concentration/time curve (AUC in mg.h/l) from 10 to 58 h were determined by the Trapezoidal method. The values for $K_m$ (mg/l) and $V_{max}$ (mg/l/day) derived from these individual curves were estimated using Michaelis-Mentin kinetics and an iterative computer programme.

*Statistics.*—Paired Student's *t* tests were used to determine the within-patient variation in the derived experimental values.

### RESULTS

The mean age, sex, body weight and diagnosis of the 8 patients are given in Table I. Six patients had metastatic melanoma, 1 had metastatic rectal carcinoma and 1 had metastatic hypernephroma.

TABLE I.—*Details of patients studied*

| Subgroup | Age (yrs) | Sex | Wt. (kg) | Diagnosis (all metastatic) |
|---|---|---|---|---|
| BCG | 42 | M | 71 | Malignant melanoma |
| | 49 | F | 47 | Malignant melanoma |
| | 46 | M | 67 | Rectal carcinoma |
| | 71 | F | 60 | Malignant melanoma |
| *C. parvum* | 70 | M | 58 | Hypernephroma |
| | 50 | F | 76 | Malignant melanoma |
| | 59 | M | 78 | Malignant melanoma |
| | 44 | M | 67 | Malignant melanoma |

The areas beneath the concentration/time curve, values for $K_m$ and $V_{max}$ are given in Table II. No within-subject difference was found in the healthy volunteers or the patients. There was, however, a significant difference in the AUC and $K_m$ values between healthy volunteers and patients (Table II).

The proportion of the administered dose excreted in the urine as p-HPPH during the first 72 h after administration is given in Table III. No difference was found between the 2 tests in healthy volunteers or patients. It was noted that the subjects who excreted the major proportion of the

TABLE II.—*Comparison of AUC (mg.h/l), $K_m$ (mg/l) and $V_{max}$ (mg/l/day) (mean±s.e.) before (Day 1) and after (Day 10) immunotherapy*

| | A.U.C. (mg.h/l) | | $K_m$ (mg/l) | | $V_{max}$ (mg/l/day) | |
|---|---|---|---|---|---|---|
| Subgroup | Day 1 | 10 | 1 | 10 | 1 | 10 |
| Control | 272±41 | 263±35 | 11·1±1·9 | 10·4±1·5 | 11·8±1·6 | 10·9±1·0 |
| BCG | **148±27 | 142±31 | *6·1±0·7 | 5·9±1·0 | 8·4±1·1 | 9·1±0·6 |
| *C. parvum* | **94±19 | 85±19 | **4·2±0·9 | 3·8±0·8 | 6·8±2·0 | 6·4±2·2 |

Significant difference between control subjects and patients receiving BCG or *C. parvum* are indicated by * ($P<0·05$) and ** ($P<0·01$) (two-tailed).

TABLE III.—*Urine excretion of 5-(p-hydroxyphenyl)-5-phenylhydantoin (% of administered dose) (mean±s.e., n=4) during the first 72 h after an oral dose of phenytoin (500 mg) before (Day 1) and after (Day 10) immunotherapy.*

| Subgroup | Day 1 | Day 10 |
|---|---|---|
| Control | 40·7±2·2 | 45·4±3·8 |
| BCG | 52·9±2·9 | 56 ±5 |
| *C. parvum* | 59·7±10·7 | 60·3±2·6 |

dose on the first day of the study did so when the test was repeated. No difference was seen between the healthy controls and the patients.

### DISCUSSION

Diphenylhydantoin was selected as the test drug because the plasma half-time correlates well with other drugs of therapeutic relevance, for example, amylobarbitone, glutethimide and sulphinpyrazone (Brien *et al.*, 1975). It was considered that phenytoin was the most suitable drug for study in this context. Though there is suggestive evidence that chronic administration of phenytoin in epileptic patients may cause immunosuppression (Sorrell *et al.*, 1971; Sorrell & Forbes, 1975), it was considered extremely unlikely that a single 500mg dose would produce immunosuppression, and there is as yet no described relationship between immunosuppression and impaired hepatic metabolism. Similarly, whilst chronic administration of phenytoin may produce enzyme induction (Petruch *et al.*, 1974) there was no evidence of this in the control patients.

The decay of serum phenytoin in man is not a simple first-order process (Arnold & Gerber, 1970). The dose-dependent decay is explained by saturation of the drug-metabolizing system, and appears to change from zero to first order as the serum concentration decreases (Bochner *et al.*, 1972). Elimination can be explained by Michaelis-Mentin kinetics (Arnold & Gerber, 1970; Gerber & Wagner, 1972) and a computer programme is available which fits the serum concentration/time curve after an oral loading dose of phenytoin (Mawer *et al.*, 1974). Ten hours following such a dose, absorption is complete and the drug is distributed within a one-compartment open model. From such a model values of $K_m$ and $V_{max}$ may be calculated.

The values so derived indicate that immunotherapy using either BCG or *C. parvum* had no statistically significant effect on the metabolism of phenytoin in the patients studied. Similarly, no difference was seen in the proportion of urinary p-HPPH excreted during the first 48 h, in both patients and healthy volunteers. One interesting observation was the significantly smaller values for AUC in the patients than in the healthy volunteers. This observation suggests that liver metabolism may be altered in patients with no clinical or biochemical evidence of metastatic hepatic infiltration, an observation that may have therapeutic relevance.

Thus, immunotherapy alone does not impair the ability of the liver to hydroxylate phenytoin and presumably, therefore, the ability to metabolize other drugs that are handled similarly. It is not possible to predict from this study whether metabolism would be impaired by repeated administration of either BCG or *C. parvum.* A different sampling schedule may have detected a change, but the 10-day interval was selected on projection from animal data (Farquhar *et al.*, 1975; Mosedale & Smith, 1975) and because peak immunostimulation occurs 10–14 days after immunotherapy (Thatcher *et al.*, 1978). There is, however, evidence that the concurrent administration of immunotherapeutic stimulants and cyclophosphamide will depress aminopyrene N-demethylation (Lipton *et al.*, 1977) and therefore, caution should be exercised when combining these 2 treatment modalities.

### REFERENCES

ARNOLD, K. & GERBER, N. (1970) The rate of decline of diphenylhydantoin in human plasma. *Clin. Pharmacol. Ther.*, **11**, 121.

BOCHNER, F., HOOPER, W. C., TYRER, J. H. & EADIE, M. J. (1972) Effect of dosage increments on blood phenytoin concentrations. *J. Neurol. Neurosurg. Psychiat.*, **35**, 873.

BRIEN, J. F., INABA, T. & KALOW, W. (1975) Comparative drug elimination in man—diphenylhydantoin and amobarbital. *Eur. J. Clin. Pharmacol.*, **9**, 79.

FARQUHAR, D., LOO, T. L., GUTTERMAN, J. U., HERSH, E. M. & LUNA, M. A. (1975) Inhibition of drug metabolizing enzymes in the rat after Bacillus Calmette-Guerin treatment. *Biochem. Pharmacol.*, **25**, 1529.

GERBER, N. & WAGNER, J. G. (1972) Explanation of dose-dependent decline of diphenylhydantoin plasma levels by fitting to the integrated form of the Michaelis-Mentin equation. *Res. Commun. Chem. Pathol. Pharmacol.*, **3**, 455

KABRA, P. M. & MARTON, L. J. (1976) High-pressure liquid chromatographic determination of 5-(4-hydroxyphenyl)-5-phenylhydantoin in human urine. *Clin. Chem.*, **22**, 1672.

LIPTON, A., HEPNER, G. W. & HARVEY, H. A. (1977) Decreased hepatic drug demethylation in patients receiving BCG and chemo-immunotherapy. *Proc. Am. Ass. Clin. Oncol.*, **18**, 276.

MACGEE, J. J. (1970) Rapid determination of diphenylhydantoin in blood plasma by gas-liquid chromatography. *Anal. Chem.*, **42**, 421.

MAWER, G. E., MULLEN, P. W., RODGERS, M., ROBINS, A. G. & LUCAS, S. B. (1974) Phenytoin dose adjustment in epileptic patients. *Br. J. Clin. Pharmacol.*, **1**, 163.

MOSEDALE, B. & SMITH, M. A. (1975) *Corynebacterium parvum* and anaesthetics. *Lancet*, i, 168.

PETRUCH, F., SCHÜPPEL, R. V. A. & STEINHILBER, G. (1974) The effect of diphenylhydantoin on hepatic drug hydroxylation. *Eur. J. Clin. Pharmacol.*, **7**, 281.

SORRELL, T. C., FORBES, I. J., BURGESS, F. R. & RISCHBIETH, R. H. C. (1971) Depression of immunological function in patients treated with phenytoin sodium (sodium diphenylhydantoin). *Lancet*, ii, 1233.

SORRELL, T. C. & FORBES, I. J. (1975) Depression of immune competence by phenytoin and carbamazepine. *Clin. Exp. Immunol.*, **20**, 273.

SOYKA, L. F., HUNT, W. G., KNIGHT, S. E. & FOSTER, JR., R. S. (1976) Decreased liver and lung drug-metabolizing activity in mice treated with *Corynebacterium parvum*. *Cancer Res.*, **36**, 4425.

THATCHER, N., SWINDELL, R. & CROWTHER, D. (1979) Effects of *Corynebacterium parvum* and BCG therapy on immune parameters in patients with disseminated melanoma—A sequential study over 28 days. II. Changes in nonspecific (NK, K and T cell) lymphocyte-toxicity and delayed hypersensitivity skin reactions. *Clin. Exp. Immunol.* (in press).